# Admission Circulating Cell-Free DNA Levels as a Prognostic Factor in Pediatric Burns

**DOI:** 10.1155/2022/5004282

**Published:** 2022-06-08

**Authors:** D. Halpern, A. Cohen, N. Sharon, Y. Krieger, E. Silberstein, T. Michael, A. Douvdevani, Y. Shoham

**Affiliations:** ^1^Joyce & Irving Goldman Medical School, Faculty of Health Sciences, Ben Gurion University of the Negev, Beer Sheba, Israel; ^2^Plastic and Reconstructive Surgery Department and Burn Unit, Soroka University Medical Center, Faculty of Health Sciences, Ben Gurion University of the Negev, Beer Sheba, Israel; ^3^Department of Public Health, Faculty of Health Sciences, Ben Gurion University of the Negev, Beer Sheba, Israel; ^4^Clinical Biochemistry and Pharmacology Department, Soroka University Medical Center, Faculty of Health Sciences, Ben Gurion University of the Negev, Beer Sheba, Israel

## Abstract

**Background:**

Burn injuries in children are a major physical and psychological trauma, often a severe condition with long-term consequences. Current methods of assessing the extent of burn injuries on admission are inaccurate. Circulating cell-free DNA (cfDNA) is a potential marker of tissue damage that may be useful in burn care.

**Objective:**

To explore the use of cfDNA admission levels as a prognostic marker of pediatric burn severity and outcome.

**Methods:**

cfDNA levels of 38 pediatric burn patients (otherwise healthy) and 12 matched pediatric controls (minor elective surgery patients) admitted to our center were quantified by a direct fluorometric assay.

**Results:**

We found significantly higher admission cfDNA levels in the patient group (median 724 ng/ml, range 44-4405), compared to the control group (median 423 ng/ml, range 206-970, Mann–Whitney, *P* = 0.03) and a significant difference between cfDNA levels of partial-thickness burns (median 590 ng/ml, range 44-2909) and full-thickness burns (median 2394 ng/ml, range 528-4405, Mann–Whitney, *P* = 0.01). We also found significant correlations between cfDNA levels and hospitalization duration (Spearman, *R* = 0.42, *P* < 0.01) and undergoing surgical procedures (Spearman, *R* = 0.40, *P* < 0.01). PICU admission did not correlate to cfDNA levels (Spearman, *R* = 0.14, *P* = NS). *Discussion.* Admission cfDNA levels may be a valuable objective tool for assessing the severity of pediatric burn injuries on admission, including correlations with the length of hospitalization and surgical burden.

**Conclusion:**

Admission cfDNA levels may be a promising novel pediatric burn assessment method. Further investigation of cfDNA levels in healthy children standardized to age and larger cohorts are needed to establish cfDNA as a valuable prognostic factor for pediatric burn injury.

## 1. Introduction

Burn injuries in children are often a severe and sometimes life-threatening condition, with physical and psychological long-term consequences [1–4]. The pediatric subpopulation constitutes a large portion of burn patients, in some studies even as high as 43% of all burn injuries [5]. The epidemiology of burn injuries depicted in various studies shows that in the pediatric population the male to female ratio is around 1.7 : 1 and scalding to be the predominant cause (59-92%), followed by flame (6-33%), electricity (0-10%), chemical (0-7%), and other causes (0-23%) [5–8]. Evaluation of the severity of burns is crucial and is the basis for the choice of treatment. Burn injuries are heterogenous, and their appearance often changes during the initial days after injury, thus making the determination of severity difficult [9]. Assessing the severity of burn injuries is usually done using subjective methods, primarily visual and tactile inspection of wound characteristics such as appearance, capillary refill, and sensibility, and therefore rely mostly on clinician experience [9, 10]. Currently, clinicians use the affected percentage of total body surface area (%TBSA) involved and burn depth. Pape et al. showed that the clinical assessment of burn depth was only 50-80% accurate compared to histological examination [10, 11]. Therefore, additional factors, preferably objective variables, are needed to assess burn severity.

In the past few years, there have been developments in quantifying cell injury and death accurately and efficiently by circulating cell-free DNA (cfDNA) levels. First, the ability to measure cfDNA concentration was established, and later, tests aimed at identifying abnormal DNA sequences in oncologic diseases and prenatal care were introduced [12–14]. Additional conditions in which cfDNA levels are elevated and of possible prognostic value include myocardial infarction, sepsis, stroke, autoimmune diseases, and trauma [15–20]. Additionally, novel methods of assessing cfDNA levels in burns demonstrated that cfDNA quantitative levels might serve as an accurate and promising tool for determining the extent of cell injury, presumably due to necrosis, apoptosis, and NETosis that frees DNA from cell nuclei to the bloodstream [21, 22]. Shoham et al. found adult patients' admission cfDNA levels to be significantly elevated compared to those of healthy controls and demonstrated statistically significant correlations between cfDNA admission levels and burn depth, total body surface area (TBSA), and total burn volume (TBV), the result of multiplying TBSA by burn depth degree [21]. Cell-free DNA levels are usually quantified using the quantitative polymerase chain reaction (PCR), which is expensive and time-consuming in comparison to the proposed alternative, a rapid fluorescent assay introduced by Douvdevani et al. [14]. This method has already been used to quantify burn injury in adults, with promising initial results and can offer a fast and cost-efficient test of utilizing cfDNA levels to assess burns in children [21].

To the best of our knowledge, the concept of assessing burn injury using circulating cfDNA levels has not been studied before in children. We believed it might be helpful as an objective method for evaluating pediatric burn patients. We hypothesized that cfDNA levels in pediatric burns will be elevated compared to healthy children and that cfDNA elevation will correspond to the severity of the burns and their outcomes.

## 2. Methods

This prospective cohort study included children admitted to the Soroka University Medical Center pediatric burns and intensive care units. Inclusion criteria were generally healthy children under 18 years old, not pregnant, admitted less than 12 hours from a burn injury, with no concomitant trauma. Patient results were compared with a group of age-matched healthy children admitted for minor elective surgery. The controls' inclusion criteria were generally healthy and not pregnant children under 18 years old. The Institutional Review Board approved the study, and legal guardians gave their written informed consent for all participating patients and controls.

### 2.1. cfDNA Analysis

Patient blood samples were obtained at admission in standard gel blood collection tubes (Vacuette, Greiner Bio-One, Frickenhausen, Germany). Blood samples were centrifuged at 2000G for 10 minutes at 4°C, and the serum was transferred to collection tubes and stored in −20°C. cfDNA levels were quantified by a direct rapid fluorometric assay, fluorochrome SYBR Gold, which does not require prior processing of samples, that is, DNA extraction and amplification. Briefly, SYBR Gold Nucleic Acid Gel Stain (Invitrogen, Paisley, UK) was diluted 1 : 1000 in dimethyl sulphoxide and then 1 : 8 in phosphate-buffered saline. Twenty microliters of serum or DNA standard were applied to a 96-well plate, and 80 microliters of diluted SYBR Gold were applied to each well. Fluorescence was measured with a 96-well fluorometer (SpectraMax Paradigm plate reader (molecular devices)) at an emission wavelength of 535 nm and an excitation wavelength of 485 nm. The method was tested compared to the gold standard QPCR and was found to have a good correlation of *R*^2^ = 0.9987 (*P* < 0.0001).

### 2.2. Burn Assessment

Burn depth was clinically assessed by plastic surgeons experienced in burn care. %TBSA was assessed by the Lund and Browder chart. Total burn volume (TBV) was calculated by multiplying %TBSA by the burn depth degree (partial-thickness burns calculated as 2nd degree and full-thickness as 3rd degree).

### 2.3. Sample Size

Based on the former report from our institute regarding adult patients [21] that showed a difference of 1000 ng/ml between burn patients and controls, we used WINPEPI (version 11.65) and calculated the sample size needed for statistical power of 80% to be 33 burn patients and 12 controls.

### 2.4. Statistical Analysis

Patient demographic and clinical data were collected, including burn characteristics, admission cfDNA levels in blood samples, PICU admission, surgical treatment, and length of hospitalization. Patient variables were collected and analyzed with SPSS (ver. 26.0). The association between the continuous outcome and independent nominal variates was tested using one-tailed Mann–Whitney tests, as well as Kruskal-Wallis test due to our hypothesis that cfDNA levels will be higher in burns involving severe tissue injury. To examine the association between the nominal variates themselves, we used the *χ*^2^ test. The correlation between cfDNA, TBV, hospitalization days, PICU admission, and the number of surgeries was evaluated using Spearman correlation test. A statistically significant difference was denoted by *P* < 0.05.

## 3. Results

Fifty children were enrolled in the study between 2018 and 2021, of them 38 patients and 12 controls.

### 3.1. Demographic and Clinical Data

The patient group included 20 males (52.6%) and 18 females (47.4%) aged 6-187 months old, and the control group included 7 males (58.3%) and 5 females (41.7%) aged 8-147 months old. There were no significant differences in age or gender between the patient and control groups (Mann–Whitney test and *χ*^2^ test, respectively, Table 1).

### 3.2. Burn Characteristics

**Figure 1:**
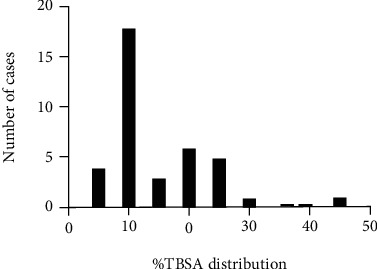
Histogram showing the patients' TBSA distribution frequency.

Most burn injuries were due to scalding (73.7%), followed by flame burns (21.1%). There was 1 chemical burn and 1 contact burn. There were 33 (86.8%) partial-thickness burns in the burn patient group and 5 (13.2%) full-thickness burns. The median TBSA was 12%, ranging from 5% to 45%. A comparison between partial-thickness and full-thickness burns is shown in Table 2. There was no significant difference between the TBSA of the partial-thickness burns and the full-thickness burns (Mann–Whitney, *P* = 0.26). TBSA distribution and comparison by burn depth are shown in Figure 1. The median number of hospitalization days was 10 (range 2-37). Nineteen burn patients were admitted to PICU (50%), and 11 were treated surgically (28.9%), ranging between 1 and 4 surgical interventions.

### 3.3. cfDNA

**Figure 2:**
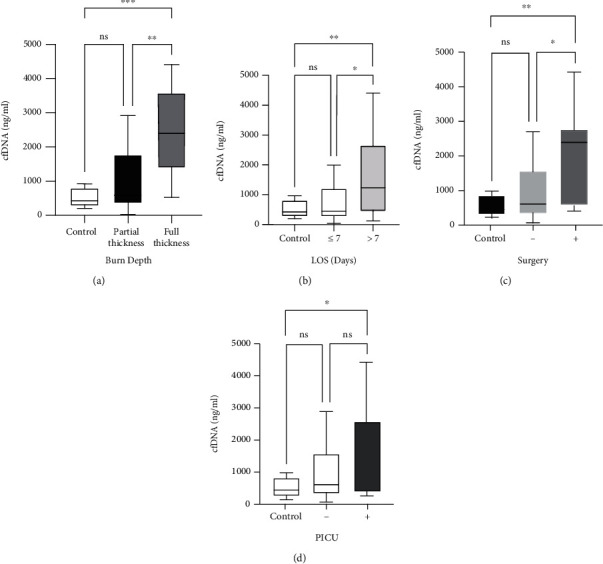
Boxplots showing cfDNA levels distribution by (a) burn depth, (b) length of stay (LOS) in the hospital, (c) surgical intervention, and (d) PICU admission. ^∗^*P* value < 0.05, ^∗∗^*P* value < 0.01, ^∗∗∗^*P* value < 0.001; ns: nonsignificant.

**Figure 3:**
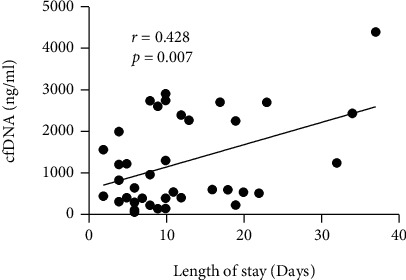
Scatter plot showing correlation between hospitalization days and cfDNA levels.

In the patient group, the median cfDNA level was 724 ng/ml (range 44-4405), and in the control group, 423 ng/ml (range 206-970). Specifically, in the partial-thickness burns group, the median cfDNA level was 590 ng/ml (range 44-2909), and in the full-thickness burns, the median was 2394 ng/ml (range 528-4405). The cfDNA levels in the patient group were significantly higher than the levels found in the control group (Mann–Whitney, *P* = 0.03). Using the Kruskal-Wallis nonparametric test to compare the controls, partial-thickness burns, and full-thickness burns, we found a significant difference in cfDNA levels within these cohorts (*H* = 7.81, *P* = 0.02, Figure 2(a)). Further investigation showed there was no significant difference between controls and partial-thickness burns (Mann–Whitney, *P* = 0.08), but there were significant differences between the controls and the full-thickness burns (Mann–Whitney, *P* < 0.01) and between the partial-thickness burns and full-thickness burns (Mann–Whitney, *P* < 0.01). There was no correlation between cfDNA levels and TBV (Spearman's *R* = 0.18, *P* = 0.13). The cfDNA levels were significantly higher in patients who stayed in the hospital for more than 7 days versus those discharged within 7 days (median 431 ng/ml versus 1234 ng/ml, Mann–Whitney, *P* = 0.04; Figure 2(b)). We also found significantly higher cfDNA levels in burn patients who underwent surgical treatment compared to those who did not undergo surgical treatment (Spearman's *R* = 0.40, *P* < 0.01; Figure 2(c)). PICU admission did not correlate with cfDNA levels (Spearman's *R* = 14, *P* = 0.19; Figure 2(d)). Additionally, we found a significant correlation between cfDNA levels and the duration of hospitalization (Spearman's *R* = 0.42, *P* < 0.01; Figure 3).

## 4. Discussion

Management of burns relies on their severity; however, as of today, there is no valid objective method for assessing the severity of burns on admission. Currently, clinicians rely mainly on clinical assessment of burn depth and TBSA using Lund and Browder charts [5, 6]. It is well recognized that this clinical assessment is subjective and inaccurate. A more accurate assessment of burn severity may lead to better guided management and prediction of outcomes.

In the past few years, there has been an increasing interest in cfDNA as a potential marker of cellular damage in general, and specifically in burn injury. Former studies demonstrated the elevation of cfDNA levels in burn patients, which is helpful since the cfDNA half-life is approximately 16 minutes, thus serving as a time-specific marker for tissue injury [12, 13, 15–19]. To the best of our knowledge, our study is the first to report cfDNA levels in pediatric burns.

In our study, the patients and controls were age- and gender-matched. The distribution of burn etiology and area were in line with the literature and with former studies showing that scalds generally cause less severe injuries than flame burns [6, 7, 23, 24].

We hypothesized that pediatric burn patients' admission cfDNA levels would be significantly elevated compared to controls. Our results indeed show significantly higher cfDNA levels in the patient group, with median cfDNA levels almost double those of the controls. This result demonstrates that admission cfDNA levels are significantly elevated in pediatric burns. Examination of the results shows an overlap in cfDNA levels of the minor partial-thickness burns and controls and a significant difference between these 2 groups as compared to the full-thickness burns. This results in , i.e., significantly higher cfDNA levels in deep burns and highlights the potential role of admission cfDNA levels as an early objective method for assessing burn depth/severity. While cfDNA levels successfully differentiated controls and minor burns from deeper burns, a significant difference between controls and minor burns was not seen. There are some possible explanations for this finding. First, the lack of a known baseline of cfDNA levels in healthy children adjusted to age. Even though the groups were age-matched, cfDNA levels were not standardized by age. Second, the variety of burn depths considered “partial thickness” makes this group very diverse in the amount of cell damage, as deep partial thickness burns lead to more overall tissue damage as compared to superficial partial thickness burns. Third, this study's relatively small number of patients makes it difficult to achieve statistical power to significantly differentiate between relatively close cfDNA levels. This is shown by the lack of significant correlations between cfDNA levels and TBV, although these correlations were demonstrated in former studies in adults [21].

We found significant correlations between cfDNA levels and the length of hospitalization stay and the need for surgical intervention. These results further highlight the potential of cfDNA levels as an early (on admission) prognostic factor that may be helpful in decision-making or planning when admitting burn patients, i.e., higher levels of admission cfDNA levels may assist in preparing a surgical intervention plan, and in preparations for a longer length of stay (e.g., social worker intervention in order to assist families with coping with a longer hospitalization, loss of parental income due to leave of absence etc.). We did not find a significant correlation between PICU admission and cfDNA levels. This finding may be explained by the fact that in our center, less severe burns are sometimes admitted to PICU for a 24-hour observation based on PICU occupancy.

Our results demonstrate admission cfDNA levels as an objective tool for assessing the severity of burn injuries, differentiating between partial-thickness and full-thickness burns, and correlating with the length of hospitalization and the need for surgical treatment. More severe burns may thus be distinguished from less severe burns by utilizing the rapid fluorometric assay we used in this study to measure cfDNA levels. The rapid fluorometric assay is faster, easier to use, and more cost-effective than the PCR method. Our findings, combined with the advantages of the rapid fluorometric assay, point to the potential of using this method in common practice in pediatric burn care in the future.

## 5. Conclusion

Our study demonstrates that admission circulating cfDNA levels appear to have value as a tool for early objective assessment of burn severity in children. There is a need for further investigation to establish circulating cfDNA levels as a valuable method of assessing burn injury in the future. Future studies should focus on standardizing the normal levels of cfDNA in healthy children adjusted to age, so cfDNA levels after cell injury may be compared to them. Additionally, larger patient and control cohorts are needed to achieve greater statistical power and more robust results.

## Figures and Tables

**Table 1:** Patients' and controls' characteristics. Nominal variables (age, gender) were compared using *χ*^2^ test, and continuous variables (cfDNA) were compared using the Mann–Whitney test.

	Patients (*n* = 38)	Controls (*n* = 12)	*P* value
Age (months) median (range)	24 (6-187)	75 (8-147)	0.24
Male gender (%)	20 (52.6%)	7 (58%)	0.61
cfDNA (ng/ml) median (range)	724 (44-4405)	423 (206-970)	0.03

**Table 2:** Comparison between partial-thickness and full-thickness burns. Nominal variables (age, gender, and burn etiology) were compared using the *χ*^2^ test, and continuous variables (TBSA, TBV, and cfDNA) were compared using the Mann–Whitney test.

	Partial-thickness (*n* = 33)	Full-thickness (*n* = 5)	*P* value
Age (months), median (range)	24 (6-187)	73 (14-168)	0.08
Male gender	14 (45%)	4(80%)	0.34
Burn etiology			<0.01
Fire	4 (12.9%)	3 (60%)	
Scald	26 (84%)	1 (20%)	
Chemical	0	1 (20%)	
Other	1 (3%)	0	
TBSA (%), median (range)	12 (5-45)	8 (6-20)	0.29
TBV median (range)	24 (10-90)	18 (16-60)	0.96
cfDNA (ng/ml), median (range)	590 (44-2909)	2394 (528-4405)	0.03

**Table 3:** Patients' demographic and clinical data.

Patient	Gender	Age (months)	Cause of burn	Burn depth	TBSA (%)	TBV	cfDNA (ng/ml)	Hospitalization days	PICU admission	Number of surgeries
1	Male	132	Fire	Partial-thickness	25	50	1234	32	Yes	2
2	Male	21	Scald	Partial-thickness	8	16	1992	4	No	0
3	Male	6	Scald	Partial-thickness	15	30	2608	9	Yes	0
4	Male	48	Scald	Partial-thickness	12	24	392	5	No	0
5	Female	119	Fire	Partial-thickness	25	50	214	19	Yes	0
6	Female	114	Scald	Partial-thickness	8	16	296	4	No	0
7	Male	42	Scald	Partial-thickness	12	24	395	12	No	0
8	Male	19	Scald	Partial-thickness	12	24	1295	10	No	0
9	Female	30	Scald	Partial-thickness	8	16	1198	4	No	0
10	Female	43	Scald	Partial-thickness	13	26	212	8	Yes	0
11	Female	13	Scald	Partial-thickness	11	22	283	6	No	0
12	Male	126	Fire	Partial-thickness	10	20	631	6	No	0
13	Female	187	Scald	Partial-thickness	25	50	590	16	Yes	0
14	Male	10	Scald	Partial-thickness	8	16	2741	8	Yes	1
15	Male	73	Scald	Partial-thickness	29	58	381	10	Yes	4
16	Female	24	Scald	Partial-thickness	14	28	44	6	No	0
17	Male	18	Scald	Partial-thickness	20	40	132	10	Yes	0
18	Female	19	Scald	Partial-thickness	10	20	94	6	No	0
19	Female	149	Fire	Partial-thickness	11	33	533	11	No	3
20	Male	24	Scald	Partial-thickness	11	22	382	7	No	0
21	Female	72	Scald	Partial-thickness	25	50	501	22	Yes	0
22	Female	18	Other	Partial-thickness	8	16	1555	2	No	0
23	Female	13	Scald	Partial-thickness	5	10	818	4	No	0
24	Male	24	Scald	Partial-thickness	21	42	585	18	Yes	1
25	Female	13	Scald	Partial-thickness	20	40	954	8	Yes	0
26	Female	20	Scald	Partial-thickness	9	18	431	2	No	0
27	Male	12	Scald	Partial-thickness	23	46	2437	34	Yes	0
28	Male	14	Scald	Partial-thickness	5	15	2909	10	No	1
29	Female	9	Scald	Partial-thickness	10	20	2705	17	No	1
30	Female	68	Scald	Partial-thickness	45	90	2700	23	Yes	0
31	Female	18	Scald	Partial-thickness	8	20	1214	5	No	0
32	Male	52	Fire	Partial-thickness	18	36	2268	13	Yes	0
33	Male	6	Scald	Partial-thickness	12	24	128	9	Yes	0
34	Male	57	Scald	Full-thickness	6	18	2746	10	Yes	2
35	Male	14	Other	Full-thickness	6	18	2394	12	Yes	4
36	Male	168	Chemical	Full-thickness	8	16	528	20	Yes	2
37	Male	167	Fire	Full-thickness	20	60	2250	19	No	0
38	Female	73	Fire	Full-thickness	20	60	4405	37	Yes	4

**Table 4:** Controls' demographic and clinical data.

Patient	Gender	Age (months)	cfDNA (ng/ml)
1	Female	85	270
2	Male	34	409
3	Male	126	970
4	Female	12	438
5	Female	66	512
6	Male	132	314
7	Male	84	325
8	Female	147	206
9	Male	23	741
10	Male	8	938
11	Female	23	211
12	Male	120	842

## Data Availability

All burn victims' data are presented in Table 3. Data regarding the controls are shown in Table 4.

## Additional Points

*Research Limitations*. It is well established that various injury mechanisms may cause an elevation in circulating cfDNA levels. We tried to overcome this issue by narrowing the inclusion criteria to otherwise healthy patients with no known reasons that may confound the results such as concomitant trauma or other chronic/acute illnesses. The lack of known baseline cfDNA levels standardized to age makes it difficult to assess the injury's direct and indirect effects on cfDNA. Moreover, the time from injury was taken into consideration by including only patients admitted within 12 hours from injury; however, we did not further standardize the results according to the time from injury. As cfDNA half-life is relatively short, this may also be a limitation [12, 13, 15–19]. Finally, we believe a study group of 50 children is not big enough to be conclusive. Therefore, we recommend that further research focus on more defined age groups and include larger patient and control cohorts.
